# Supplementary material for: Predicting antibody kinetics and duration of protection against SARS-CoV-2 following vaccination from sparse serological data
Source: PLoS Comput Biol. 2025 Jun 18;21(6):e1013192. doi: 10.1371/journal.pcbi.1013192 (PMC12193770; doi:10.1371/journal.pcbi.1013192)
Supplement: S1 Appendix — (PDF) [file pcbi.1013192.s001.pdf]

S1 Appendix.

Predicting antibody kinetics and duration of protection against SARS-CoV-2 following vaccination from sparse serological data

Julia Deichmann*, Noam Barda, Michal Canetti, Yovel Peretz, Yael Weiss-Ottolenghi, Yaniv Lustig, Gili Regev-Yochay, Marc Lipsitch

* jdeichmann@hsph.harvard.edu

Supplementary Methods

S1 Training and test data

We used serum samples from 75 % of the study population as a training data set for model inference and used samples from the remaining 25 % as a test data set for the evaluation of model predictions. As the majority of participants did not present any comorbidities, we ensured that the training and test split resulted in groups with similar characteristics. We first divided the population into groups with and without comorbidities. Next, we randomly assigned 75 % of individuals in the group without comorbidities to the training and 25 % to the test set. We proceeded similarly for assigning individuals from the group with comorbidities in order to have data from participants with underlying conditions present in both data sets.

The characteristics of the full study population as well as summaries for the training and test sets are provided in Table 1 in the main text, with characteristics remaining similar between the training and test sets.

S2 Statistical model and inference

S2.1 Observation model

We assume that the observed antibody levels y_{ij} of individual i at time j are log-normally distributed with

$$y_{ij} \sim LogN(A_{ij}, \sigma),$$

where A_{ij} represent the modeled antibody levels. The measurement error σ is estimated with the remaining parameters during model inference and prior $\sigma \sim N_+(0, 0.1)$.

Antibody levels are imputed for censored observations above the limit of quantification, y_{max}. Similar to uncensored observations, we assume that the imputed values $\hat{y}_{ij}$ follow a log-normal distribution around the model predicted values, and enforce that they fall above y_{max}. This implies that

$$\begin{aligned} \hat{y}_{ij} &\sim LogN(A_{ij}, \sigma), \\ \text{with} \quad \hat{y}_{ij} &> y_{max}, \quad \text{if} \quad y_{ij} > y_{max}. \end{aligned}$$

S2.2 Individual-level parameters

The antibody kinetics model comprises nine immunological parameters that need to be estimated. We use mixed effects models to infer individual-level parameter values and capture the variability between study participants in the corresponding immunological processes, while estimating the distribution of these parameters across the entire

population. For the antibody boost following the first and second dose, gB_1 and gB_2, respectively, and the decay rates of short-lived, c_s, and long-lived antibody-producing cells (APCs), c_l, these mixed effects models take on the general form:

$$\begin{aligned} log(p_i) &= log(p_{pop}) + X_i^T \cdot \beta_p + \epsilon_{p,i} \qquad && \epsilon_{p,i} \sim N(0, \sigma_p) \\ &= log(p_{pop}) + X_i^T \cdot \beta_p + u_{p,i} \cdot \sigma_p \qquad && u_{p,i} \sim N(0,1), \end{aligned} \tag{1}$$

where p_i represents parameter gB_1, gB_2, c_s or c_l of individual i. The intercept term $log(p_{pop})$ describes the average parameter value p_{pop} across the whole population. The fixed effects term $X_i^T \cdot \beta_p$ captures deviations from the population average that can be explained by individual characteristics, summarized in a k-dimensional covariate vector X_i, with slope vector β_p that is common between all individuals. Finally, the remaining inter-individual variability is captured in the random effects term $u_{p,i} \cdot \sigma_p$, which consists of the individual deviation $u_{p,i}$ in standard deviations σ_p.

Age, BMI and sex (female=0, male=1) are added as fixed effects to the covariate matrix X. In addition, we include the comorbidities listed in Table 1 in the main text. We assume a log-log relationship between the continuous covariates age and BMI and the model parameters with fixed effects terms $\beta_p^{age} \cdot log(age/43)$ and $\beta_p^{BMI} \cdot log(BMI/25)$, respectively. Hence, the population parameters are defined for a typical female of 43 years with a BMI of $25\,\mathrm{kg/m^2}$ and no comorbidities.

We assume that there are no systematic differences in initial antibody levels between individuals, since all participants have a negative anti-SARS-CoV-2 IgG assay before entering the study. We therefore removed fixed effects for parameter A_0. Similarly, we assume that antibodies have a decay rate r that is consistent across the population. Consequently, differences in the waning of antibody levels will be captured in the decay rates of APCs. Since ρ describes a proportion and is bound between 0 and 1, we define $\rho_i = logit^{-1}(\rho_{pop} + u_{\rho,i} \cdot \sigma_\rho)$.

We fix the delay in antibody response following vaccination at $\delta_1 = 10$ days for dose one and $\delta_2 = 4$ days for dose two. This is based on the time we first see an increase in the mean change in antibody levels across individuals compared to their pre-vaccination serology, or similarly compared to their serology during receipt of the second vaccine dose. While we allow the antibody boost gB_j and delay δ_j to be different for each vaccine dose j, we assume that ρ and all decay rates remain the same, since potential differences cannot be inferred due to the proximity of the two vaccination events relative to processes with longer time scales involved in the humoral response.

S2.3 Priors

We define the following priors for population- and individual-level parameters:

$$\begin{aligned} log(A_{0,pop}) &\sim N(log(0.05), 0.5) \\ log(gB_{1,pop}) &\sim N(log(0.8), 0.2) \\ log(gB_{2,pop}) &\sim N(log(10), 0.2) \\ \rho_{pop} &\sim N(3, 0.1) \\ log(r) &\sim N(log(0.033), 0.1) \\ log(c_{s,pop}) &\sim N(log(0.14), 0.1) \\ log(c_{l,pop}) &\sim N(log(0.002), 0.5) \\ \beta_{[gB1/gB2/cs/cl]} &\sim N(0, 0.2) \\ u_{[A0/\rho/gB1/gB2/cs/cl]} &\sim N(0, 1) \\ \sigma_{[A0/\rho/gB1/gB2/cs/cl]} &\sim N_+(0, 0.3) \end{aligned} \tag{2}$$

Parameters A_0, gB_1 and gB_2 are related to the amplitude of the antibody response and are therefore assay-specific. We use a prior on ρ that corresponds to a high proportion of short-lived APCs [1, 2]. We set priors for rate parameters based on antibody and APC half-lives. IgG antibodies have a half-life of $t_{1/2} = 21$ days [3], which relates to their decay rate via $r = log(2)/t_{1/2}$. Short-lived APCs have a half-life of only a few days [4]. Here, we set the prior according to $t_{1/2} = 5$ days. The half-life of long-lived APCs, on the other hand, remains uncertain and we choose a wider prior for the corresponding rate parameter.

S2.4 Inference

We sampled from the posterior distributions using the No-U-Turn sampler (NUTS) [5], a Markov Chain Monte Carlo (MCMC) algorithm implemented in Stan [6]. We used 1 000 warm-up iterations, 2 000 sampling iterations and four chains. No divergences were detected and estimated parameters had $\hat{R} \leq 1.03$, indicating that chains have mixed well. Processing of results was performed in R with the CmdStanR package.

S3 Prediction of antibody levels on test data

Next, we predict the anti-SARS-CoV-2 antibody response of a new individual that was not part of the training data set based on limited serological data. We consider study participants from the test data set and provide one IgG measurement per individual for different sampling times between M1 and M6.

To predict antibody levels, we first update the model parameters given the new data. As before, model parameters p (Eq. 1) are defined as

$$log(p_i) = log(p_{pop}) + X_i^T \cdot \beta_p + u_{p,i} \cdot \sigma_p \qquad u_{p,i} \sim N(0, 1),$$

consisting of a population average, fixed and random effects. For the population-level parameters p_{pop}, β_p and σ_p, we re-use the posterior samples generated on the training data set ($n = 8\,000$), and we insert covariate values X_i of individual i from the test data set. The random effects, however, contain an unknown individual-level deviation $u_{p,i}$. First, we draw 8 000 samples of the deviation $u_{p,i}$ for each parameter and individual from a standard normal distribution to generate the complete individual-level posterior samples for p_i and simulate the corresponding IgG antibody trajectories. These represent predictions for study participants in the test data set without model adjustment.

Next, we select parameter realizations among the posterior samples that generate predictions $A_i(t_i)$ such that the available observation y_i provided at time t_i falls into the 90 % posterior prediction interval of the observation model, with $y_i \sim LogN(A_i, \sigma)$. The selected parameter combinations and resulting antibody trajectories are what we consider the updated model.

Model predictions are evaluated using the root mean squared log error (RMSLE) based on all observations available for an individual. In addition to the general prediction accuracy, we are also interested in the accuracy of forward predictions over varying time horizons and their dependence on the timing of serological testing after vaccination. Since testing was performed monthly after administration of the second vaccine dose, we provide a sample from month one (M1: days 39–63 following the first dose [7]), two (M2: days 64–91), three (M3: days 92–119), four (M4: days 120–147), five (M5: days 148–175) or six (M6: days 176–203) for model adjustment and analyze the RMSLE of the predicted antibody response separately for the subsequent months. However, not every individual in the test data set had serological testing performed each

month. We therefore consider an individual in the analysis of a specific month only if a corresponding sample is available.

S4 Duration of protection

Moreover, we examine the ability of the model to predict the duration of protection provided by the vaccine. We define an anti-SARS-CoV-2 IgG antibody level of 500 BAU/mL, corresponding to a sample-to-cutoff ratio of 11.6 in our data [8], as the threshold for protection. This threshold was previously determined for protection against the delta variant in a study among vaccinated household members exposed to an index case [9]. The probability of a contact becoming infected dropped to 11% if the contact's baseline IgG antibody concentration was higher than 500 BAU/mL. We evaluate the time at which simulated antibody levels fall below the threshold and report the median duration of protection for each individual.

We consider individuals that have a median duration of protection larger than 0 as protected, meaning that at least 50 % of sampled antibody trajectories cross the protective threshold. The remaining individuals are considered to remain unprotected following vaccination.

S4.1 Validation

For participants in the test data set, we compare our predictions against the observed duration of protection. Initially, we validate our categorization into protected and unprotected. Afterwards, we validate the predicted duration for protected individuals. To do so, we evaluate whether our prediction falls into the time interval between the last data point above and the first data point below the protective threshold during the waning phase of the antibody response. Note that we include participants only if this time interval does not exceed 45 days to avoid overestimating the prediction accuracy of our model due to sparse serological sampling.

References

1. Slifka MK, Antia R, Whitmire JK, Ahmed R. Humoral Immunity Due to Long-Lived Plasma Cells. Immunity. 1998;8(3):363–372. doi:10.1016/S1074-7613(00)80541-5.

2. Yman V, White MT, Asghar M, Sundling C, Sondén K, Draper SJ, et al. Antibody responses to merozoite antigens after natural Plasmodium falciparum infection: kinetics and longevity in absence of re-exposure. BMC Medicine. 2019;17(1):22. doi:10.1186/s12916-019-1255-3.

3. Sompayrac L. How the immune system works. 6th ed. John Wiley & Sons Ltd; 2019.

4. Khodadadi L, Cheng Q, Radbruch A, Hiepe F. The Maintenance of Memory Plasma Cells. Frontiers in Immunology. 2019;10:721. doi:10.3389/fimmu.2019.00721.

5. Hoffman MD, Gelman A. The No-U-turn sampler: adaptively setting path lengths in Hamiltonian Monte Carlo. Journal of Machine Learning Research. 2014;15(1):1593–1623.

6. Stan Development Team. Stan Modeling Language Users Guide and Reference Manual 2.36; 2024.

7. Levin EG, Lustig Y, Cohen C, Fluss R, Indenbaum V, Amit S, et al. Waning Immune Humoral Response to BNT162b2 Covid-19 Vaccine over 6 Months. New England Journal of Medicine. 2021;385(24):e84. doi:10.1056/NEJMoa2114583.

8. Lustig Y, Gonen T, Meltzer L, Gilboa M, Indenbaum V, Cohen C, et al. Superior immunogenicity and effectiveness of the third compared to the second BNT162b2 vaccine dose. Nature Immunology. 2022;23(6):940–946. doi:10.1038/s41590-022-01212-3.

9. Regev-Yochay G, Lustig Y, Joseph G, Gilboa M, Barda N, Gens I, et al. Correlates of protection against COVID-19 infection and intensity of symptomatic disease in vaccinated individuals exposed to SARS-CoV-2 in households in Israel (ICoFS): a prospective cohort study. The Lancet Microbe. 2023;4(5):e309–e318. doi:10.1016/S2666-5247(23)00012-5.
