# Supplementary figures and images for: Predicting antibody kinetics and duration of protection against SARS-CoV-2 following vaccination from sparse serological data
Source: PLoS Comput Biol. 2025 Jun 18;21(6):e1013192. doi: 10.1371/journal.pcbi.1013192 (PMC12193770; doi:10.1371/journal.pcbi.1013192)

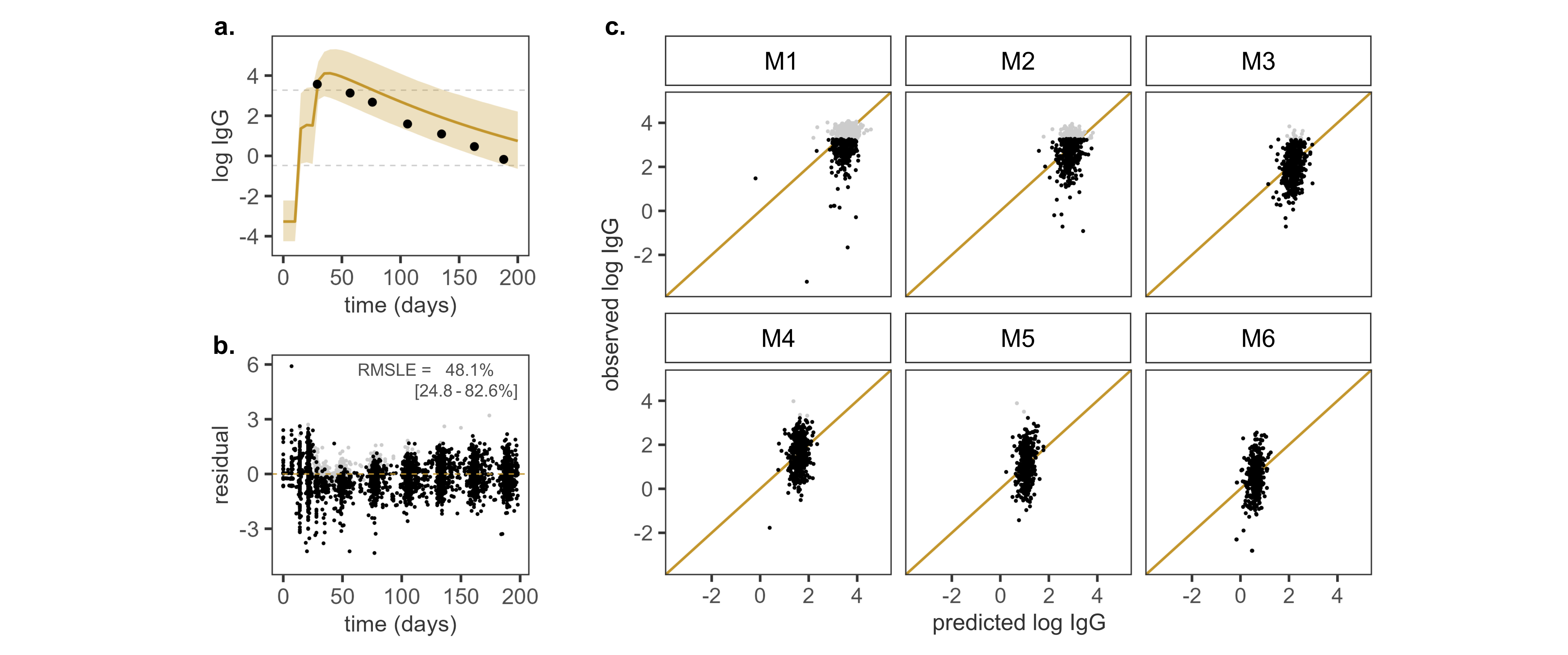

Supplement: S3 Fig — (a) Predicted IgG antibody trajectory (yellow) for one example individual from the test data set without model adjustment. The solid line represents the geometric mean prediction, the shaded area indicates the 90% posterior predictive interval. (b) Residuals and the RMSLE for antibody levels after receipt of the second vaccine dose (median, IQR) are shown for the full test population. (c) Observed vs predicted antibody levels by time of observation between M1 and M6, where geometric mean predictions display lower inter-individual variability than the observed data. Lighter shades are used for measurements above the limit of quantification. (TIF) [file pcbi.1013192.s004.tiff]
